# Molecular Characterization of the Porcine Group A Rotavirus NSP2 and NSP5/6 Genes from São Paulo State, Brazil, in 2011/12

**DOI:** 10.1155/2013/241686

**Published:** 2013-07-15

**Authors:** Bruna Rocha Passos Barbosa, Nara Thiers Cacciatori Galleti Bernardes, Laila Andreia Rodrigues Beserra, Fábio Gregori

**Affiliations:** Department of Preventive Veterinary Medicine and Animal Health, Faculty of Veterinary Medicine, University of São Paulo, Avenida Prof. Dr. Orlando Marques de Paiva 87, Cidade Universitária, 05508-270 São Paulo, SD, Brazil

## Abstract

Rotaviruses are responsible for the acute diarrhea in various mammalian and avian species. The nonstructural proteins NSP2 and NSP5 are involved in the rotavirus replication and the formation of viroplasm, cytoplasmic inclusion bodies within which new viral particles morphogenesis and viral RNA replication occur. There are few studies on the genetic diversity of those proteins; thus this study aims at characterizing the diversity of rotavirus based on NSP2 and NSP5 genes in rotaviruses circulating in Brazilian pig farms. For this purpose, 63 fecal samples from pig farms located in six different cities in the São Paulo State, Brazil, were screened by nested RT-PCR. Seven strains had the partial nucleotide sequencing for NSP2, whereas in six, the total sequencing for NSP5. All were characterized as genotype H1 and N1. The nucleotide identity of NSP2 genes ranged from 100% to 86.4% and the amino acid identity from 100% to 91.5%. For NSP5, the nucleotide identity was from 100% to 95.1% and the amino acid identity from 100% to 97.4%. It is concluded that the genotypes of the strains circulating in the region of study are in agreement with those reported in the literature for swine and that there is the possibility of interaction between human and animal rotaviruses.

## 1. Introduction

 Group A rotaviruses (GARV) are the major causes of viral diarrhea in a variety of animal young species worldwide [1]. In pig farms, they are responsible for economic losses due to death of animals, poor growth performance, and costs of diagnostic and treatment [2, 3]. Porcine GARVs are associated with weaning and postweaning enteritis in piglets and more often detected in piglets between 1 and 8 weeks of age [4, 5].

 Rotavirus belongs to the family Reoviridae, subfamily Sedoreovirinae, and its genome consists of 11 segments of double-stranded RNA (dsRNA), which encode six structural proteins (VP1, VP2, VP3, VP4, VP6, and VP7) and six nonstructural proteins (NSP1–NSP6), with all genes being monocistronic except for segment 11 which encodes two proteins, NSP5 and NSP6 [6].

 Encoded by segment 8 (1059 bp), NSP2 is a 35 kDa protein which assembles into octamers and is composed of 317 amino acids [7]. NSP2 has a role in packaging and replication, performing activities of nonspecific binding to single-stranded RNA (ssRNA) to start the synthesis of dsRNA, translocation of viral RNA during packaging, and helicase ATP-independent, triphosphatase, and NDP kinase activities [7–10]. 

 NSP5 encoded by segment 11 (667 bp) is a 21 kDa protein that is hyperphosphorylated and O-glycosylated, and it consists of 198 amino acids, with abundance of serine (21%) and threonine (4.5%) [11–13]. NSP5 interacts with NSP2 to form cytoplasmic structures known as viroplasms, inside of which RNA replication and morphogenesis of new viral particles occur [1, 14, 15]. The interaction with NSP2 also enhances the process of NSP5 hyperphosphorylation [9, 16]. The segment 11 also contains the coding sequence of NSP6 in a second ORF, whose function is still unknown, but it preliminarily interacts with NSP5 in dimerization and hyperphosphorylation processes. However, given the low levels of expression of NSP6, this suggests that this protein did not have an essential regulatory role [17–19].

 Traditionally, GARVs are classified on the basis of two outer capsid proteins (VP4 and VP7), which induce the formation of neutralizing antibodies, with the VP7 determining the genotype G, whereas VP4 determines P genotype [1, 6]. However, a new classification for rotavirus was recently proposed which takes into account all 11 genomic segments, and the genotype N was determined for NSP2 protein and genotype H for NSP5 [20]. So far, nine genotypes N (N1 to N9) and 11 genotypes H (H1 to H11) have been defined. In humans, genotypes N1, N2, and N3 and H1, H2, and H3 have been described, whereas in pigs, H1 and N1 have been identified [5, 20]. As observed for VP4 and VP7, NSP2 and NSP5 also share genotypes between humans and animals, as previously reported by Martella et al., Matthijnssens et al., Tsugawa and Hoshino, and Khamrin et al. [5, 21–23].

 The zoonotic aspect and interspecies transmission of rotavirus have been described by several authors [5, 21, 24, 25], and in this context the swines are considered as a major source of infection and important reservoirs for genetic and antigenic diversity of human rotaviruses [26, 27].

 Despite the importance of genetic characterization of rotavirus circulating strains, data on the NSP2/5/6 molecular characterization in pigs in Brazil are scarce. Therefore, the objective of this study was to sequence the NSP2 and NSP5/6 genes of group A porcine rotavirus isolated from different pig herds in the São Paulo State, Brazil, in order to understand the phylogenetic relationships among them, as well as regarding other strains previously isolated from several regions around the world. 

## 2. Materials and Methods

### 2.1. Samples

 A total of 63 fecal samples from 34-day-old piglets with and without symptoms of diarrhea were used to study the NSP2 and NSP5 genes of GARV. The samples were collected in pig farms located in six different cities in the state of São Paulo, Brazil, from 2011 to 2012. 

 The rotavirus strain NCDV was used as positive control and ultrapure water previously treated with 0.1% diethyl pyrocarbonate (DEPC water) (Invitrogen) as negative control.

### 2.2. Detection of Rotavirus

 For initial screening, the viral RNA was extracted from fecal samples using the TRIzol Reagent method (Invitrogen) according to the manufacturer's instructions, followed by nested RT-PCR technique as described by Salem et al. [28]. The extracted RNA was denatured at 95°C for 5 minutes and then subjected to RT-PCR. 

### 2.3. RT-PCR

 The reverse transcription reaction (cDNA synthesis) was performed using the following protocol. Briefly, 7 *μ*L of extracted RNA was added to a mixture containing 1x First Strand Buffer (Invitrogen), dNTP (1 mM each), 10 mM DTT, 1 mM Random Hexamers (50 ng/*μ*L, Invitrogen), 200 U MMLV Reverse Transcriptase (Invitrogen), and ultrapure DEPC-treated water for a final reaction volume of 20 *μ*L. The RT was performed at 37°C for 1 h, followed by incubation at 70°C for 15 min.

 The amplification of the NSP2 partial gene (975 bp) was performed using four primers, one of them described by Matthijnssens et al. [21] and others developed in the present study based on alignments of sequences retrieved from GenBank (Table 1). For NSP5/6, the complete segment of 667 bp was amplified using the primers previously described by Matthijnssens et al. [21] (Table 1).

 Thus, the PCR reaction consisted of adding 5 *μ*L of cDNA to a mixture containing 1x PCR Buffer (Invitrogen), dNTP (0.2 mM each), 0.2 *μ*M of each primer (Table 1), 2 mM MgCl_2_, 5 U of Taq DNA Polymerase (Invitrogen), and ultrapure DEPC-treated water for a final reaction volume of 25 *μ*L. The PCR was performed at 94°C/3 min, followed by 40 amplification cycles (94°C/45 s, 45°C/45 s, and 70°C/2,5 min) and a final extension of 70°C for 7 min. 

### 2.4. DNA Sequencing

 The amplicons were purified with ExoSAP-IT (Affymetrix) according to the manufacturer's instructions and sequenced in both directions using the BigDye Terminator Cycle Sequencing Kit v. 3.1 (Life Technologies) followed by the application of the BigDye Xterminator kit (Life Technologies) for the removal of unincorporated DNA terminators, also according to the manufacturer's instructions, on an automated DNA sequencer ABI-3500 (Applied Biosystems). 

### 2.5. Phylogenetic Analysis

 The sequences obtained were edited using Bioedit v. 7.1.3.0 program [29], and multiple alignments were done using the Clustal W v. 2.1 software [30]. The nucleotide and deduced amino acid sequences of NSP2 and NSP5/6 genes were compared with those of reference strains available at NCBI (National Center for Biotechnology Information) GenBank database using the BLAST (Basic Local Alignment Search Tool) server [31]. 

 Phylogenetic analyses were conducted using MEGA version 5.1 [32], and the phylogenetic tree was built using the neighbor-joining method (1,000 bootstrap trials) with maximum composite likelihood as substitution model. 

### 2.6. Nucleotide Sequence Accession Numbers

 The nucleotide sequences of NSP2 and NSP5/6 genes of the rotavirus strains from this study have been deposited in GenBank under the accession numbers KC117140 to KC117146 for NSP2 and KC117147 to KC117152 for NSP5. 

## 3. Results

### 3.1. Detection of Rotavirus

 Seventeen of the 63 fecal samples (17/63 or 27%) tested positive for rotaviruses by nested RT-PCR [28] from five different cities (5/6). 

### 3.2. Analysis of NSP2 Gene Sequences

 We sequenced a partial NSP2 fragment (975 bp or 97%) in 7 of the 17 positive strains analyzed (SWRV1 to SWRV7). 

**Figure 1:**
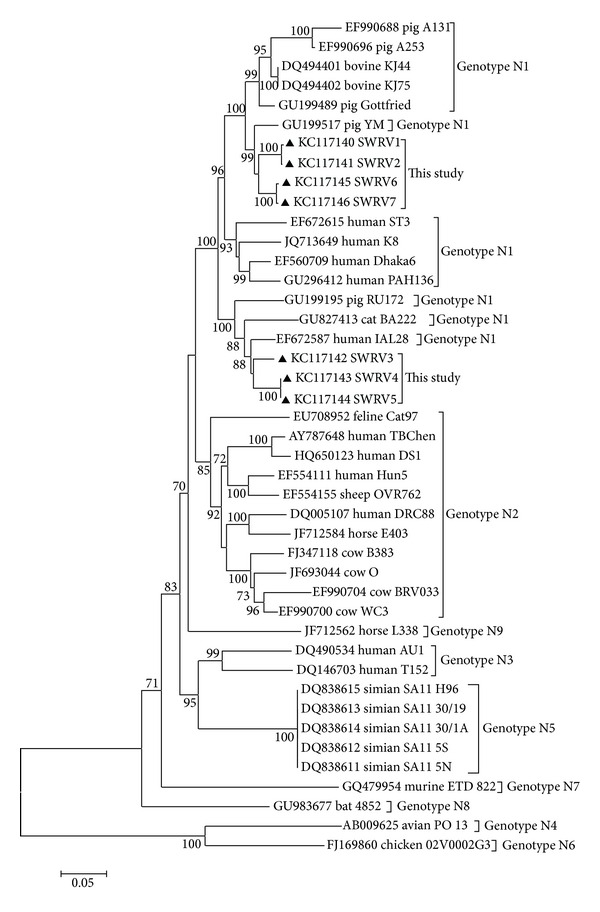
Phylogenetic tree constructed with the neighbor-joining method using maximum composite likelihood as substitution model for a partial region (975 bp) of the NSP2 gene of the group A porcine rotavirus of the strains SWRV1 and SWRV7 with others representing human and animal GARVs. The scale represents the number of substitutions per site. Only bootstrap values >70% are shown. Pig strains defined in the present study are preceded by black triangles, and they are identified as accession number/host/strain/NSP2 genotype.

 Comparing the nucleotide and predicted amino acid sequences of the strains of the present study with others already deposited in GenBank, the data revealed that SWRV 1, 2, 6, and 7 were more closely related to the porcine rotavirus strain, YM, in terms of nucleotides (94.7–95.4%) and to bovine strains, KJ44 and KJ75, in terms of amino acid (98.0–98.3%). With regard to the strains SWRV 3, 4, and 5, they shared maximum identities with the human rotavirus strain, IAL28 (94.0–95.1% in terms of nucleotide and 96.1% for amino acids). The phylogenetic tree revealed that the strains clustered together with the N1 genotype (Figure 1).

 The motifs related to the functions of translocation of RNA (75 bases corresponding to the 5′ noncoding region 3′ and the beginning of ORF), to the NTPase (histidine at position 225), and to the cysteine residues 6, 8, 85, and 285, which are involved in the formation of disulfide bridges [10, 33, 34], were conserved between the samples of this study.

 There was variability in the RNA-binding domain of NSP2 corresponding to amino acid residues 205–241 (A207S, R215K, I218V and S216F, and I218A) [10, 33].

### 3.3. Analysis of NSP5/6 Gene Sequences

 Complete sequences of these genes and their corresponding proteins were obtained in 6 of the 17 positive strains analyzed (6/17) (SWRV1 to SWRV6). 

**Figure 2:**
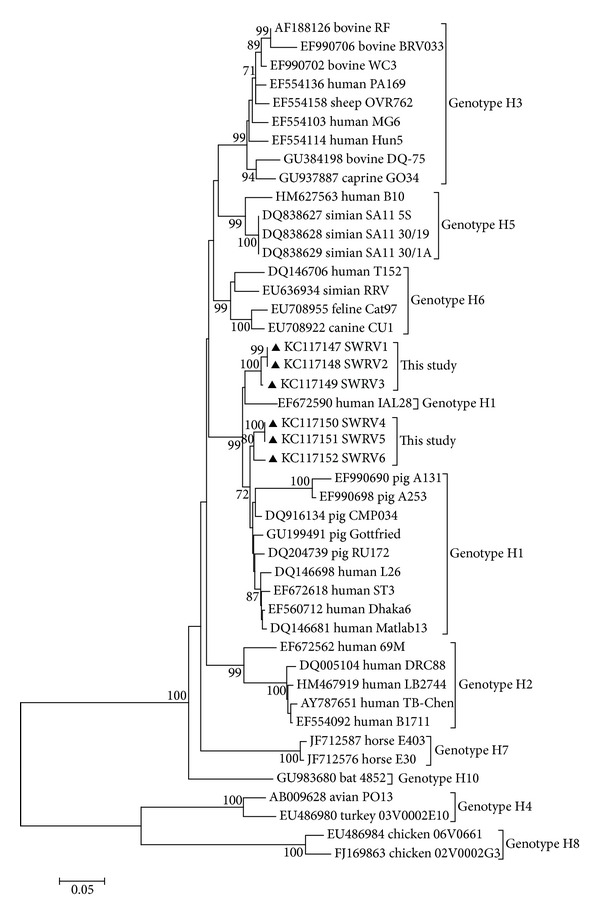
Phylogenetic tree constructed with the neighbor-joining method using maximum composite likelihood as substitution model for the NSP5 complete gene of the group A porcine rotavirus of the strains SWRV1 and SWRV6 with others representing human and animal GARVs. The scale represents the number of substitutions per site. Only bootstrap values >70% are shown. Pig strains defined in the present study are preceded by black triangles, and they are identified as accession number/host/strain/NSP5 genotype.

 The comparison among the nucleotide and putative amino acids sequences of the strains of this study with others from human and animal previously deposited in GenBank revealed a high identity among SWRV 1, 2, and 3 and porcine strains Gottfried, RU172 and CMP034 (94.0–95.1% nucleotide and 96.1% amino acid identity). With respect to SWRV 4, 5, and 6, they also shared maximum identities with the porcine rotavirus strains, Gottfried and CMP034 (97.1%, in terms of nucleotide and 99.4% for amino acids). The phylogenetic tree revealed that the strains clustered together with the H1 genotype (Figure 2).

 For NSP6, in terms of amino acids, the strains SWRV 1, 2, and 3 shared maximum identities values from 95.6% with human strains 2007744509, 2008747100, and 2007719698, while SWRV 4, 5, and 6 were most closely related to porcine strain, PRG942 (98.9–100%).

 The nine phosphorylation sites of NSP5 concerning serine residues at positions 2, 4, 30, 37, 42, 56, 67, 101, 127, and 163 [35] present conserved between the samples of this study, except for the residue at position 37 (S37N).

## 4. Discussion

Studies carried out about the occurrence of RVA in pigs from different Brazilian regions have shown a relatively wide distribution of this agent in the creations around the country [36–38]. Five of the six cities studied presented samples positive for rotavirus at an overall prevalence of 27% (17/63), very similar to that found by Gregori et al. [36] of 29.9%.

 Our study revealed the circulation of genotype N1 of NSP2 in the cities sampled, which, for pigs, is the only N genotype reported so far, but that has also been described in humans, cattle, and cats [5, 20, 39]. Likewise, the genotype H1 detected for NSP5 has been reported in pigs, which also occurs in humans and animals [5, 20]. It should be noted that no genotypic classification was defined for NSP6, since this protein ORF is entirely contained within the NSP5 ORF. Therefore, as suggested by Matthijnssens et al. [39], the phylogenetic analysis of both would be nearly identical.

 The NSP2 genes for the strains SWRV 3, 4, and 5 were the closest related to that of the IAL28 strain, a G5 human rotavirus strain isolated in Brazil in 1992 from a child with diarrhea, and that G5 is another genotype commonly described in pigs [40]. Although the highest nucleotide and amino acid identity values for NSP5 had been with porcine rotavirus strains, phylogenetic tree deduced from this gene revealed that the strains SWRV 1, 2, and 3 segregated in a cluster that also included the human strain IAL28 with which those strains showed high nucleotide and amino acid identities ranging from 94.1 to 94.6% and 94.3%, respectively (Figure 2). The findings of genetic relationships of the porcine strains of this study with those of human rotavirus, as well as detection of genotypes common to both species, suggest the occurrence of interspecies transmission, in which the pigs likely act as a source of infection for humans [24, 39].

 Several studies corroborated our findings by reporting high degrees of genetic similarity between human and animal strains, as well as the occurrence of N1 and H1 genotypes in human strains (5, 21, 27, 41). Esona et al. showed that in six G8 human African strains, the genes VP7, NSP2, and NSP5 had high identity with animal strains (83–99% and 97–99% nucleotide and amino acid values, resp.) [41]. Additionally, Ghosh et al. determined the nucleotide sequence of the genes encoding VP1, VP2, VP3, and NSP1, NSP2, and NSP3 of the porcine strain, RU172, being that NSP2 clustered in a group predominantly consisting of human rotavirus [42].

 It has been shown that NSP2 motif related to the RNA binding consists of the amino acid residues 205–241, while the cysteine residues 6, 8, 85, and 285 are involved in disulfide bridges which ensure the octameric structure of this protein [10, 33]. Furthermore, these studies also reveal an extensive conserved region of 75 bases (b) including 35b in the 5′ noncoding region 3′ and the first 30b of the ORF as a region in which the conformational NSP2 changes occur, leading to translocation of ssRNA. The NTPase activity of NSP2 is related to amino acid histidine at position 225, which acts as a catalyst residue for enzymatic function of this protein, so that the mutation in this residue prevents the synthesis of dsRNA [34]. Thus, mutations in regions concerning the binding domain of NSP2 with the RNA, R215K, S216F, and I218V, found in the strains of the present study in relation to the standard strain Gottfried (Accession number GU199489), have been reported in samples standard OSU, UK, NCDV, Wa, and SA11 [33, 43, 44]. On the other hand, for mutations at positions I218A and A207S, there are no reports in the literature.

 In regard to NSP5, nine phosphorylation sites were identified in this protein concerning serine residues at positions 2, 4, 30, 37, 42, 56, 67, 101, 127, and 163 [35]. Compared to the standard strain Gottfried (Accession number GU199491), the strains of this study showed a mutation from serine to asparagine at position 37 which does not seem to be significant since some isoforms of this protein may not provide the phosphorylation sites at residues 30 and/or 37, and then, serine would be absent at these sites, but without altering the phosphorylation process, as demonstrated by Sotelo et al. [35].

 The data showed low rates of genetic variability for NSP2 and NSP5/6 in the strains of the present study. Indeed, these proteins are generally more stable because of structural and functional constraints and lower exposure to immune selection [45]. 

 More genetic characterization studies would be helpful for detection of interspecies transmission [21, 27, 46]; however, only collection and analysis of RV specimens collected in close geographic and temporal proximity in a prospective manner, as shown by Steyer et al. [25], will provide firm evidence. Thus, considering that there are few studies in Brazil aiming at molecular characterization of NSP2 and NSP5 in pigs, it becomes imminent monitoring swine herds and human populations in order to detect and/or prevent such situations of possible cross-infection [25].

## 5. Conclusion

 The molecular analyses performed in this study indicated that most of the samples of rotaviruses circulating in pig herds of the São Paulo State present genotypes consistent with those described in the literature for Brazil and in other countries.

## Figures and Tables

**Table 1:** Primers designed for amplification and sequencing of the NSP2 and NSP5/6 genes from the samples of this study. bp: base pairs.

Gene	Primer	Primer sequence (5′→3′)	Amplicon (bp)	Reference
NSP2	GEN-NSP2F	GGCTTTTAAAGCGTCTCAG	728	[21]
	NSP2-RW707	GTACCATTTCCAGTGATRTCTC		Developed in this study
	NSP2-FW707	CATGGTAAAGGTCACTAYAGAG	247	Developed in this study
	NSP2-RW975	ACAGTTGACTATCTTTYTACCT		

NSP5	GEN-NSP5F	GGCTTTTAAAGCGCTACAG	667	[21]
	GEN-NSP5R	GGTCACAAAACGGGAGT		
